# Comparative analysis of radiomics and deep-learning algorithms for survival prediction in hepatocellular carcinoma

**DOI:** 10.1038/s41598-023-50451-3

**Published:** 2024-01-05

**Authors:** Felix Schön, Aaron Kieslich, Heiner Nebelung, Carina Riediger, Ralf-Thorsten Hoffmann, Alex Zwanenburg, Steffen Löck, Jens-Peter Kühn

**Affiliations:** 1 Institute and Polyclinic for Diagnostic and Interventional Radiology, Faculty of Medicine and University Hospital Carl Gustav Carus, TU Dresden, Dresden, Germany; 2 OncoRay‑National Center for Radiation Research in Oncology, Faculty of Medicine and University Hospital Carl Gustav Carus, TU Dresden, Helmholtz-Zentrum Dresden-Rossendorf, Dresden, Germany; 3 Department of Visceral, Thoracic and Vascular Surgery, Faculty of Medicine and University Hospital Carl Gustav Carus, TU Dresden, Dresden, Germany; 4 National Center for Tumor Diseases (NCT/UCC) Dresden, Dresden, Germany; 5 German Cancer Research Center (DKFZ), Heidelberg, Germany

**Keywords:** Outcomes research, Cancer imaging, Hepatocellular carcinoma, Biomarkers

## Abstract

To examine the comparative robustness of computed tomography (CT)-based conventional radiomics and deep-learning convolutional neural networks (CNN) to predict overall survival (OS) in HCC patients. Retrospectively, 114 HCC patients with pretherapeutic CT of the liver were randomized into a development (n = 85) and a validation (n = 29) cohort, including patients of all tumor stages and several applied therapies. In addition to clinical parameters, image annotations of the liver parenchyma and of tumor findings on CT were available. Cox-regression based on radiomics features and CNN models were established and combined with clinical parameters to predict OS. Model performance was assessed using the concordance index (C-index). Log-rank tests were used to test model-based patient stratification into high/low-risk groups. The clinical Cox-regression model achieved the best validation performance for OS (C-index [95% confidence interval (CI)] 0.74 [0.57–0.86]) with a significant difference between the risk groups (p = 0.03). In image analysis, the CNN models (lowest C-index [CI] 0.63 [0.39–0.83]; highest C-index [CI] 0.71 [0.49–0.88]) were superior to the corresponding radiomics models (lowest C-index [CI] 0.51 [0.30–0.73]; highest C-index [CI] 0.66 [0.48–0.79]). A significant risk stratification was not possible (p > 0.05). Under clinical conditions, CNN-algorithms demonstrate superior prognostic potential to predict OS in HCC patients compared to conventional radiomics approaches and could therefore provide important information in the clinical setting, especially when clinical data is limited.

## Introduction

Hepatocellular carcinoma (HCC) is the most common primary malignant liver tumor, accounting for approximately 75% in total^1^. Overall, primary liver tumors are the second leading cause of cancer deaths worldwide with a 5-year survival rate of 18.1%^2^. The Barcelona Clinic Liver Cancer (BCLC) staging system is the most widely used algorithm in western countries to recommend prognostic prediction and first-line treatment based on tumor burden, liver function and health status of the patient^3^. Nevertheless, BCLC classification remains controversial and has limited predictive power^4,5^.

In recent years, the focus of medical research and clinical practice has shifted towards individualized medicine. Therefore, prediction of overall survival (OS) in HCC patients is of increasing importance to individually adapt potential therapy patterns and their influence on OS. Rapid advances in technology have made conventional, feature-based radiomics and deep-learning-based approaches particularly suitable for attaining these goals. Previous studies reported positive results for predicting OS of HCC patients using conventional radiomics and deep-learning algorithms^6,7^. Nevertheless, the suitability for clinical routine is questionable and despite the great potential of these technologies, a prospective transfer into clinical routine remains challenging. Patients may have received imaging for initial tumor staging in different medical centers, resulting in a large heterogeneity of acquisition parameters. Currently, there are no models for predicting OS across all HCC tumor stages and therapies. Moreover, to the best of our knowledge, it is uncertain which modelling approach, conventional radiomics or deep-learning-based approaches, is robust against the heterogeneity often encountered in clinical settings. There is evidence that conventional radiomics approaches seem more susceptible to interference, while deep-learning approaches might be more robust^8^. In our exploratory study, we therefore aimed to examine the comparative robustness of computed tomography (CT)-based conventional radiomics and deep-learning convolutional neural networks (CNN) algorithms to predict OS in HCC patients against two important sources of heterogeneity in real-world clinical settings: varied acquisition parameters and diverse tumor stages and treatments.

## Materials and methods

### Ethical aspects

The study was approved by the local ethics committee (EK 39012022) and conforms to the Declaration of Helsinki. The informed consent was waived by the ethics committee due to the retrospective nature of the study.

### Study population

A total of 343 patients with initial diagnosis of HCC were discussed between January 2010 and October 2021 in the tumor board of our University Hospital. Subsequently, patients were selected according to the following inclusion criteria:

(1) HCC patients who received a contrast-enhanced CT scan of the liver (consisting of at least an arterial and venous contrast phase) before therapy initiation; (2) the diagnosis of HCC had to be confirmed by a second imaging modality (e.g. ultrasound or magnetic resonance imaging) showing typical HCC changes, or by histopathological findings, according to the German HCC guideline^9^; (3) initial therapy and at least one follow-up imaging was carried out at our hospital.

The exclusion criteria were: (1) incomplete CT scans (missing arterial or venous contrast phase); (2) CT scans with severe artifacts; (3) patients with another active tumor disease (defined as tumor diagnosis or therapy within 2 years prior to inclusion in the present study).

**Figure 1:**
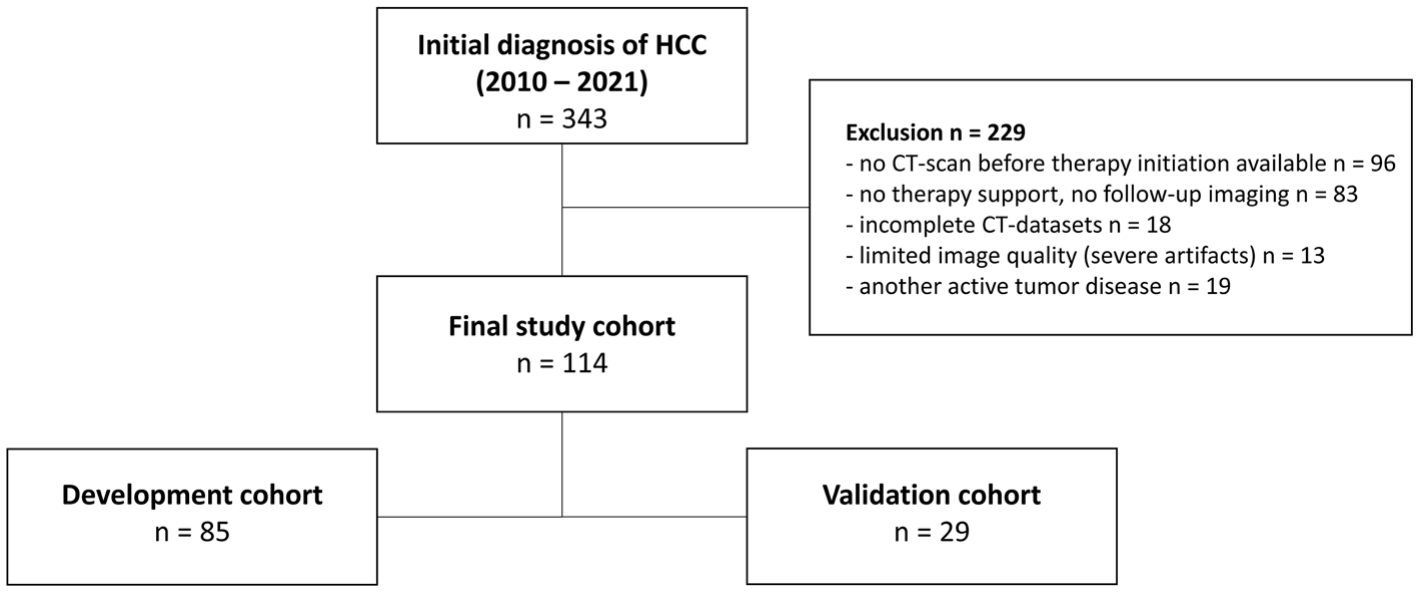
Study population. After applying inclusion and exclusion criteria, 114 patients were included and divided into a development and validation cohort by stratified randomization.

Based on these criteria (Fig. 1), a total of 114 patients were retrospectively enrolled and divided into a development (n = 85) and a validation (n = 29) cohort by stratified randomization, with stratification being performed on the initial therapy concept. Overall, the diagnosis of HCC was confirmed histopathologically in 60/114 patients, with the remaining 54/114 HCCs confirmed by imaging patterns.

### Clinical variables and radiological characteristics

Demographic data and routine lab tests were obtained for all patients. This included age, gender, time to death or follow-up time, as well as the serological parameters [alpha-fetoprotein (AFP), alanine-aminotransferase (ALAT), aspartate-aminotransferase (ASAT), albumin, total bilirubin, creatinine, gamma-glutamyltransferase (GGT) and International Normalized Ratio (INR)].

Tumor characteristics (number of lesions, presence of metastases, volume and density values of the largest HCC lesion), imaging features (status of liver cirrhosis and ascites) and initial therapy concepts [surgical resection (RES), radiofrequency ablation (RFA), liver transplantation (TRANS), transarterial chemoembolization (TACE), radiotherapy (RT), systemic therapy (ST) and best supportive care (BSC)] were recorded in addition. The ALBI-, Child–Pugh-, MELD-Score and the BCLC stage were calculated using the respective established formulae and flow charts^3,10–13^. The Child–Pugh Score was only evaluated in patients with suspected cirrhosis. Status of liver cirrhosis (present/absent) was assessed on CT by two residents (2 and 3 years of experience in liver imaging) analogously to Nebelung et al.^14^ using the following criteria: hypertrophy of the liver segments I/II/III with concomitant atrophy of the segments VI/VII, surface and parenchymal nodularity of the liver, heterogeneous density values, portal vein enlargement, and ascites. Ascites was classified as absent, mild or moderate. In case of disagreement, the final decision was made by a senior radiologist with more than 15 years of experience in liver imaging. Hepatic encephalopathy was not considered since its assessment is subjective and was not adequately documented.

### Overall survival

Overall survival was calculated as the period from the initial CT scan to the time of either death or last contact to our hospital (e.g. follow-up examination or discharge from inpatient stay).

### Imaging protocol and annotation

The CT scans were acquired on a total of 24 different scanners at 21 medical centers. Seventy-eight patients (68%) received their initial CT at our hospital. External CT scans of the remaining 36 patients (32%) were transmitted to our institution as part of routine clinical practice. Common contrast media methods (for arterial contrast: bolus tracking or approximately 25 to 35 s after contrast agent injection; for venous contrast: approximately 60 to 70 s after contrast agent injection) were applied for image acquisition. See Supplementary Table S1 for the variability of more scan parameters.

A resident with two years of experience in liver imaging contoured the liver parenchyma and the largest HCC-lesion in both contrast phases using the open-source software 3D Slicer (http://www.slicer.org)^15^. All segmentations were verified by the same resident 4 weeks later. An example of segmentation is shown in Supplementary Fig. S1.

### Standardization of the CT datasets

Variations in the circulatory capacity of patients, contrast medium injection parameters, and imaging time contribute to interindividual flood points of contrast agent^16^. The resulting differences across patients may influence the radiomics data derived from these scans^17^. To address this, a self-developed standardization procedure was performed.

For each patient, the mean CT number (*CTN*_mean_) in a circular segmentation within the aorta at the level of the coeliac trunk in the arterial and venous phase was recorded. The mean CTN for each phase (*CTN*_mean,cohort_) was used to scale the CTN of each patient (*CTN*_mean,i_) using the formula ${CTN}_{{\text{new}},\mathrm{ i}}={CTN}_{{\text{old}},\mathrm{ i}}\times \frac{{CTN}_{{\text{mean}},\mathrm{ cohort}}}{{CTN}_{{\text{mean}},\mathrm{ i}}}$.

To compensate for different slice thicknesses, all CT images were interpolated to an isotropic voxel size of 1.0 mm^3^. An anti-aliasing filter was applied, and contours were re-segmented to density values between − 200 and 500 Hounsfield units (HU). Details see Supplementary Table S2.

### Conventional radiomics risk modelling

Radiomics features were extracted from the segmentations of the liver and HCC in the arterial (*_art*) und venous (*_ven*) phase. The extraction was implemented according to the recommendations by the Image Biomarker Standardization Initiative (IBSI) using the publicly available open-source Medical Image Radiomics Processor (MIRP)^18,19^. Feature values obtained from the venous phase images were subtracted from the corresponding arterial phase values to quantify differences between both phases (*_diff*). In summary, six feature subgroups were extracted (*HCC_art, HCC_ven, HCC_diff, liver_art, liver_ven, liver_diff*), resulting in 1146 imaging features per patient.

To develop conventional radiomics models, the “Fully Automated Machine Learning with Interpretable Analysis of Results” (FAMILIAR, version 1.2.0) framework (https://github.com/alexzwanenburg/familiar) was used^20^. The utilized settings for feature extraction and model building can be found in Supplementary Table S3. Three primary models were constructed to predict OS, consisting of a clinical model, an image-based radiomics model and a combined model of clinical and imaging data. Four supplementary models analyzing the imaging segmentations separately were additionally created to compare the predictive power for OS in the different contrast phases and imaging components (whole liver parenchyma vs. HCC).

For each model, feature importance was evaluated using a 15-times repeated threefold cross-validation scheme, resulting in 45 internal models in total. In each iteration, multiple feature processing steps were applied: missing value imputation, feature transformation, filtering and clustering. The overall importance of a feature was assessed by its occurrence within the top five highest ranked features in all 45 internal models. The signature size was assigned as the median signature size of all 45 internal models. The features with the highest importance were used to create a Cox proportional hazards model for the prediction of OS. Subsequently, the models were validated on the validation cohort. Details of feature processing and model development are given in Supplementary Table S3.

### Deep-learning-based risk modelling

All segmentations of the liver and the HCC were considered. To accommodate for the large range of sizes observed for liver and HCC lesions across the image datasets, a cropping procedure was applied: all images were cropped to the 95th percentile of the distribution of liver or HCC sizes in each direction. In addition, all images were resampled to a voxel size of 2 × 2 × 2 mm^3^. The resulting image dimensions were 64 × 64 × 64 and 132 × 144 × 132 voxels for the HCC and the liver segmentations, respectively. The voxel intensities were rescaled to the interval [0, 1]. To avoid overfitting on characteristics outside of the ROI, these regions were masked by setting all voxel intensities outside the ROI to zero.

For clinical data, missing values were imputed using the median value over all patients. If the percentage of missing features for one patient exceeded 30%, the patient was excluded. All clinical data were converted to a numerical scale. The features were transformed using Yeo-Johnson normalization and Z-standardization and mapped linearly to the interval 0 to 1 based on the development cohort. Transformation parameters were applied to the validation cohort unchanged.

Four primary deep-learning-based models were constructed to predict OS, consisting of a clinical model, two image-based models (based on the HCC and liver segmentations) and a combined model of clinical and HCC imaging data. As deep-learning algorithms require significantly more computing power, it was not possible to create an imaging model consisting of all CT data as in the conventional radiomics approach. Four supplementary models analyzing the imaging segmentations separately were additionally created to compare the predictive power for OS in the different contrast phases and imaging components (whole liver parenchyma vs. HCC).

All models were implemented using the Python-based deep-learning library Pytorch^21^. The general architecture of the networks was designed after Hosny et al., Starke et al., and Nie et al. and is illustrated in Supplementary Fig. S2^22–24^. For example, the proposed image-based model consists of four convolutional layers and three fully connected layers. To regulate the model, batch normalization and dropout layers are incorporated. CT images of both arterial and venous phases form the input of the model. They are processed through the convolutional layers before being concatenated and further processed by the fully connected layers. According to Katzman et al., the loss function is set to the negative log of the Cox partial likelihood with regularizations^25^. Therefore, the final output is a single value representing the predicted hazard of the model. Details regarding the utilized hyperparameters can be found in Supplementary Table S4**.**

The number of training epochs was determined through a 15-times repeated threefold cross-validation, resulting in 45 internal models in total. Each model was trained for 500 epochs on the training fold and monitored for testing fold performance after every epoch. Model performance was assessed by the average performance of the last five epochs to reduce statistical noise. Finally, for validation, 45 models were trained on the entire development cohort using the number of epochs with the highest cross-validation performance. The final prediction for a patient was established by taking the average prediction of all 45 models.

### Evaluation of prognostic performance

Prognostic performance was evaluated by the concordance index (C-index) and the ability to stratify patients into risk groups based on the model predictions. The C-index measures the agreement between the actual OS and the model predictions. A C-index of 0.5 indicates no prognostic value, while a value close to 1 indicates perfect prediction. Patients were allocated into a low- or high-risk group for death based on the hazard values predicted by the models. The median value of these predictions was used as a cutoff on the development cohort. Patients with a predicted hazard exceeding the cutoff were assigned to the high-risk group. The difference between the low- and high-risk group was assessed using the log-rank test. The significance level was set to α = 0.05. The confidence intervals (CI) for the internal cross-validation were calculated by analyzing the distribution of the 45 model performances. To estimate the CIs for the validation, the percentile bootstrap method was performed^26^. To compare the prognostic performance of two models, a two-sample bootstrap test was employed: The difference in C-indices was computed for 1000 bootstrap samples of the validation cohort. The smaller proportion of bootstrap samples in which the C-index difference was either greater than 0 or less than 0 was multiplied by 2 to obtain the p-value.

## Results

### Study population

Development and validation cohort were balanced in terms of clinical parameters and baseline demographics (p > 0.05; Table 1).

**Table 1:** Patient characteristics of the development and validation cohort.

Variable	Development cohort (n = 85)			Validation cohort (n = 29)			p-value
	Median	Range	Missing (%)	Median	Range	Missing (%)	
Time to death of dead patients, years	1.65	0.01–6.20	n/a	1.83	0.29–3.70	n/a	0.36
Follow up time of patients alive, years	1.63	0.32–9.54	n/a	1.99	0.41–9.53	n/a	0.62
Age, years	71.11	48.17–82.12	0 (0)	70.08	39.60–84.27	0 (0)	0.84
AFP, ng/ml	8.6	0.9–707,760.0	10 (12)	5.6	1.0–22,169.6	3 (10)	0.15
ALAT, μmol/s L	0.58	0.19–4.89	3 (4)	0.57	0.27–1.78	1 (3)	0.47
ASAT, μmol/s L	0.78	0.19–3.17	5 (6)	0.71	0.38–3.33	1 (3)	0.12
GGT, μmol/s L	3.04	0.29–28.17	3 (4)	2.28	0.53–14.58	1 (3)	0.40
Albumin, g/L	39.9	22.7–48.2	7 (8)	39.9	29.3–47.0	4 (14)	0.68
Bilirubin, μmol/L	15.2	4.5–122.1	5 (6)	12.3	2.5–81.6	1 (3)	0.37
INR	1.16	0.91–2.91	4 (5)	1.14	0.93–3.15	2 (7)	0.56
Creatinine, μmol/L	79	45–162	3 (4)	87	53–141	1 (3)	0.28
MELD Score	10	7–20	7 (8)	10	7–21	2 (7)	0.88
HCC Volume arterial phase, cm^3^	18.9	0.6–1339.2	0 (0)	12.8	0.8–1473.9	0 (0)	0.83
HCC Volume venous phase, cm^3^	18.3	0.5–1246.8	0 (0)	14.0	1.0–2000.8	0 (0)	0.88
HCC Mean CTN arterial phase, HU	69.5	32.1–154.2	0 (0)	63.1	35.9–111.1	0 (0)	0.37
HCC Mean CTN venous phase, HU	74.9	34.8–124.6	0 (0)	74.5	43.1–115.5	0 (0)	0.49
HCC Variance CTN arterial phase, HU^2^	365	84–1881	0 (0)	358	127–1748	0 (0)	0.98
HCC Variance CTN venous phase, HU^2^	318	103–974	0 (0)	292	123–714	0 (0)	0.74

	Number of patients (%)		Missing (%)	Number of patients (%)		Missing (%)	
Patients lost in follow-up	40 (47)		n/a	17 (59)		n/a	0.45
Died from treatment-related causes	1 (1)		n/a	1 (3)		n/a	0.43
Sex, male/female	67/18 (79/21)		0 (0)	27/2 (93/7)		0 (0)	0.14
Therapy concept, curative/palliative	49/36 (58/42)		0 (0)	17/12 (59/41)		0 (0)	1.00
Therapy, BSC/ST/RES/RFA/RT/TACE/TRANS	5/8/25/26/6/14/1 (6/9/29/31/7/16/1)		0 (0)	2/3/8/9/2/5/0 (7/10/28/31/7/17/0)		0 (0)	1.00
Liver Cirrhosis, present/absent	60/25 (71/29)		0 (0)	21/8 (72/28)		0 (0)	1.00
Ascites, absent/mild/moderate	61/13/11 (72/15/13)		0 (0)	19/8/2 (66/28/7)		0 (0)	0.34
Child–Pugh score, 0/A/B/C	25/35/15/3 (29/31/18/4)		7 (8)	8/12/5/1 (28/41/17/3)		3 (10)	1.00
BCLC stage, A/B/C/D	29/36/17/3 (34/42/20/4)		0 (0)	12/6/10/1 (41/21/34/3)		0 (0)	0.17
ALBI score, 1/2/3	44/30/4 (52/35/5)		7 (8)	15/10/0 (52/34/0)		4 (14)	0.51
Number of HCC lesions, 1/2/3/ > 3	53/13/3/16 (62/15/4/19)		0 (0)	17/5/4/3 (59/17/14/10)		0 (0)	0.19
Lymph nodes metastases, present/absent	1/84 (1/99)		0 (0)	1/28 (3/97)		0 (0)	1.00
Distant metastases, present/absent	2/83 (2/98)		0 (0)	2/27 (7/93)		0 (0)	0.57

The variables describing the CTN, and volume refer to the largest HCC lesion. P-values were obtained by using Chi-square homogeneity tests and two-sided Mann–Whitney *U* tests for categorical and numerical variables, respectively.

*AFP* alpha-fetoprotein, *ALAT* alanine-aminotransferase, *ASAT* aspartate-aminotransferase, *BCLC* Barcelona clinic liver cancer; *BSC* best supportive care, *CTN* computed tomography number, *GGT* gamma-glutamyltransferase, *HCC* hepatocellular carcinoma, *INR* international normalized ratio, *RES* surgical resection, *RFA* radiofrequency ablation, *RT* radiotherapy, *ST* systemic therapy, *TACE* transarterial chemoembolization, *TRANS* liver transplantation.

### Conventional radiomics approach

Three primary models were developed: a clinical model, a radiomics model including all imaging features and a model combining clinical and imaging data. In addition, four supplementary radiomics models (*HCC_art; HCC_ven; liver_art; liver_ven*) were developed based on the individual image segmentations.

The median signature sizes were three, six and seven for the clinical, image-based, and combined model, respectively, and ranged between two and five for the supplementary models. For the clinical model, six patients (four in development cohort and two in validation cohort) were excluded from the analysis due to missing values > 30%. The final Cox-regression models are reported in Table 2 for the primary analyses and in Supplementary Table S5 for the supplementary analyses. The results of the internal cross-validation are shown in Supplementary Table S6.

**Table 2:** Final signatures of the primary clinical, image-based, and combined multivariate Cox-regression models and their respective parameters.

Primary conventional models	Variables	Hazard ratio [95% CI]	p-value
Clinical model	GGT	1.61 [1.15–2.27]	0.01*
	AFP	1.24 [0.92–1.67]	0.15
	volume HCC_ven	1.17 [0.81–1.68]	0.41
Image-based model	stat_rms_liver_ven	0.72 [0.52–0.99]	0.05
	szm_szhge_3d_fbn_n32_liver_diff	1.71 [1.17–2.52]	0.01*
	morph_moran_i_liver_diff	0.69 [0.49–0.96]	0.03*
	dzm_lde_3d_fbn_n32_liver_diff	1.26 [0.90–1.78]	0.18
	morph_pca_least_axis_liver_art	1.66 [1.13–2.45]	0.01*
	morph_pca_least_axis_liver_diff	1.31 [0.92–1.87]	0.14
Combined model	GGT	1.58 [1.11–2.23]	0.01*
	stat_p90_liver_ven	0.88 [0.61–1.28]	0.51
	szm_szhge_3d_fbn_n32_liver_diff	1.70 [1.11–2.60]	0.02*
	dzm_lde_3d_fbn_n32_liver_diff	1.18 [0.83–1.67]	0.35
	stat_max_HCC_diff	1.30 [0.92–1.83]	0.14
	ih_min_grad_fbn_n32_liver_ven	0.92 [0.62–1.36]	0.56
	morph_pca_least_axis_liver_diff	1.17 [0.80–1.72]	0.42

The hazard ratio (HR) [95% CI] and the corresponding p-values of the regression are shown based on the development cohort.

*AFP* alpha-fetoprotein, *art* arterial phase, *diff* difference, *CI* confidence interval, *GGT* gamma-glutamyltransferase, *HCC* hepatocellular carcinoma, *ven* venous phase.

*Statistically significant (p < 0.05).

**Figure 2:**
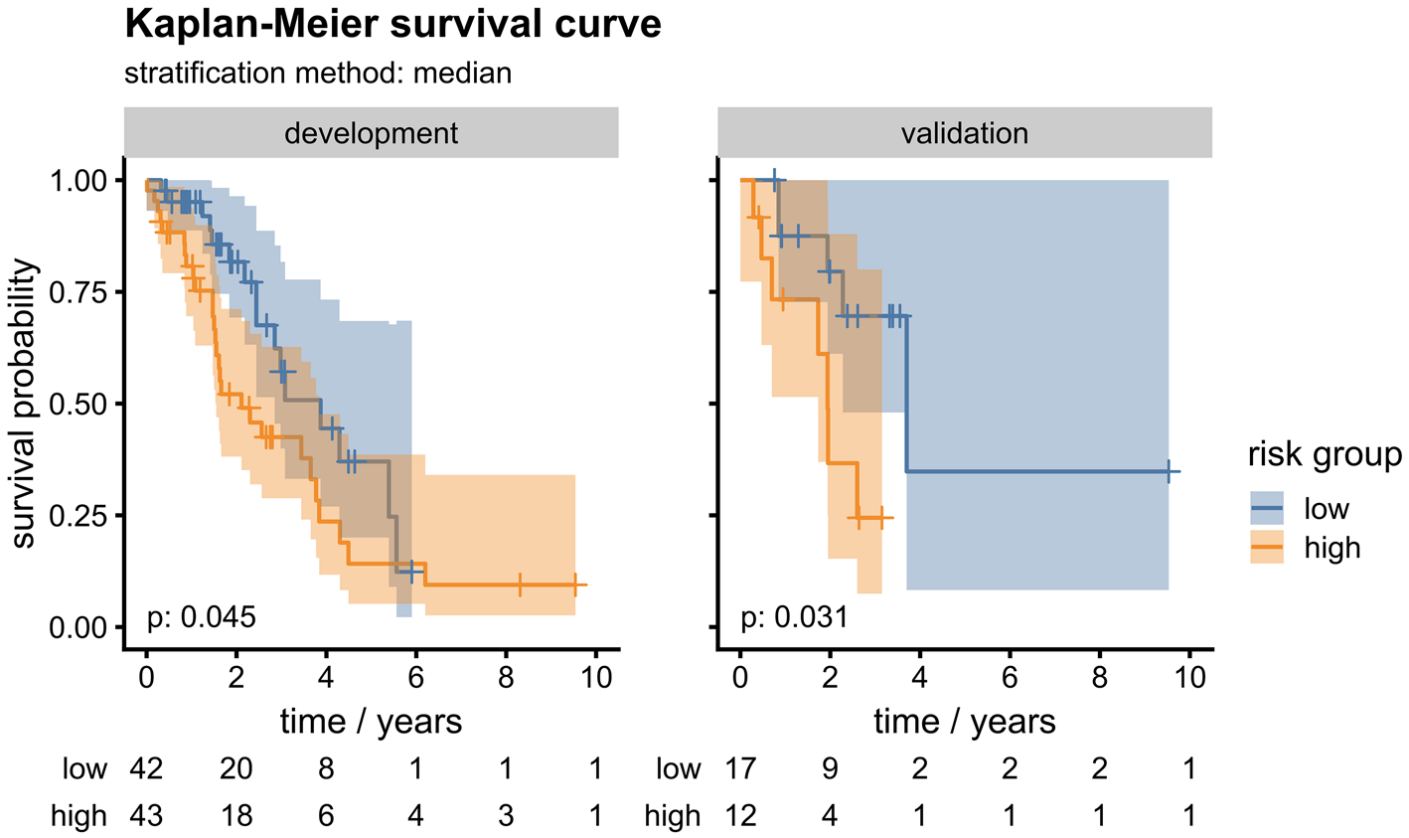
Kaplan–Meier survival curves of patients stratified into risk groups (cutoff value = 1.024 years) by the clinical model in the development and validation cohort. Differences in OS between low- and high-risk groups were evaluated by the log-rank test.

In independent validation (Table 3), the clinical model showed the best result with a C-index [95% CI] of 0.74 [0.57–0.86], outperforming the image-based and combined model significantly (p = 0.016 and p = 0.034, respectively). The best supplementary model (*HCC_art*) clearly outperformed the primary image-based model, which showed a performance close to random prediction (C-index [95% CI] 0.66 [0.48–0.79] vs. C-index [95% CI] 0.51 [0.30–0.73]). The risk stratification into high- and low-risk groups showed significant differences in OS only for the clinical model (p = 0.031; Fig. 2).

**Table 3:** Final performance of the Cox-regression models in development and independent validation: C-indices [95% CI] and p-values for risk stratification.

		Development cohort		Validation cohort	
		C-index [95% CI]	p-value	C-index [95% CI]	p-value
Primary conventional models	Clinical model	0.69 [0.58–0.78]	0.045*	0.74 [0.57–0.86]	0.031*
	Image-based model	0.75 [0.68–0.81]	< 0.001*	0.51 [0.30–0.73]	0.66
	Combined model	0.75 [0.65–0.83]	< 0.001*	0.55 [0.40–0.69]	0.89
Supplementary conventional models	*HCC_art*	0.63 [0.53–0.73]	0.58	0.66 [0.48–0.79]	0.69
	*HCC_ven*	0.62 [0.52–0.71]	0.24	0.53 [0.36–0.71]	0.57
	*liver_art*	0.65 [0.54–0.76]	0.14	0.54 [0.30–0.74]	0.46
	*liver_ven*	0.71 [0.61–0.80]	0.013*	0.46 [0.23–0.67]	0.62

*art* arterial phase, *CI* confidence interval, *HCC* hepatocellular carcinoma, *ven* venous phase.

*Statistically significant (p < 0.05).

### Deep-learning approach

Four primary models were developed: a clinical model, two image-based models (*HCC_art* + *HCC_ven* and *liver_art* + *liver_ven*) and a model combining clinical data and HCC imaging data. Additionally, four supplementary image-based models were established (*HCC_art; HCC_ven; liver_art; liver_ven*). The results of the internal cross-validation for all primary and supplementary models are shown in Supplementary Table S7. The obtained number of epochs was 430, 39, 14 and 383 for the clinical model, the HCC-based model, the liver-based model and the combined model, respectively.

In validation (Table 4), the image-based HCC model showed the best performance of all primary models (C-index [95% CI] 0.69 [0.44–0.84]), while the clinical model was the worst (C-index [95% CI] 0.58 [0.40–0.76]). Overall, the supplementary image-based model *HCC_art* performed even slightly better than the primary HCC model (C-index [95% CI] 0.71 [0.49–0.88]). Risk stratification in groups at high and low risk of death revealed no significant results in the validation cohort (p > 0.05).

**Table 4:** Final performance of the deep-learning-based models in development and independent validation: C-Indices [95% CI] and p-values for risk stratification.

		Development cohort		Validation cohort	
		C-index [95% CI]	p-value	C-index [95% CI]	p-value
Primary deep-learning models	Clinical model	0.74 [0.69–0.81]	< 0.001*	0.58 [0.40–0.76]	0.92
	Image-based model HCC (*HCC_art* + *HCC_ven*)	0.60 [0.50–0.69]	0.45	0.69 [0.44–0.84]	0.42
	Image-based model liver (*liver_art* + *liver_ven*)	0.72 [0.63–0.80]	< 0.001*	0.65 [0.37–0.86]	0.40
	Combined model	0.65 [0.57–0.72]	0.029*	0.62 [0.41–0.81]	0.18
Supplementary deep-learning models	*HCC_art*	0.60 [0.50–0.70]	0.54	0.71 [0.49–0.88]	0.42
	*HCC_ven*	0.66 [0.57–0.75]	0.078	0.63 [0.39–0.83]	0.18
	*liver_art*	0.73 [0.62–0.81]	< 0.001*	0.63 [0.43–0.79]	0.86
	*liver_ven*	0.68 [0.58–0.80]	0.037*	0.65 [0.39–0.80]	0.17

*art* arterial phase, *CI* confidence interval, *HCC* hepatocellular carcinoma, *ven* venous phase.

*Statistically significant (p < 0.05).

### Comparison of the conventional radiomics and the deep-learning approach

**Figure 3:**
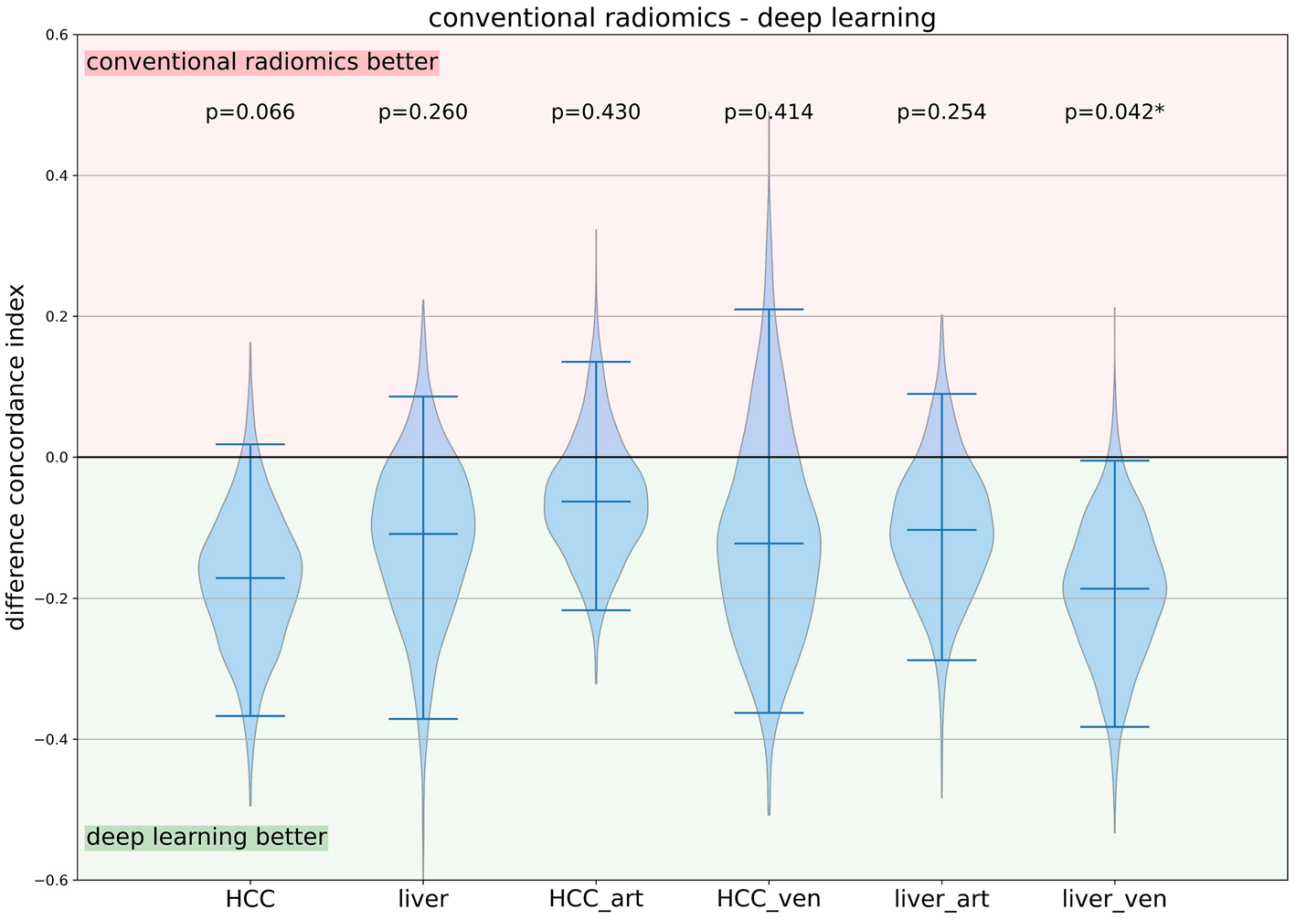
Comparison of C-indices between the conventional radiomics and the deep-learning models in the validation cohort. Positive values indicate better performance of the conventional approach, whereas negative values indicate better performance of the deep-learning approach. The whiskers represent the 95% confidence interval. The horizontal line within the distributions illustrates the median value. For the comparison of the HCC and liver models, the primary radiomics model was used. *Statistically significant (p < 0.05).

The deep-learning approach outperformed the conventional radiomics approach, with a significant improvement for the *liver_ven* model (p = 0.032). Figure 3 highlights the differences of the C-indices in the validation cohort between the conventional radiomics and deep- learning models. Figure S3 shows the calibration plots of the best performing image-based models from both the conventional radiomics and deep-learning approaches.

## Discussion

In the present study, we investigated whether conventional radiomics and deep-learning algorithms can predict OS in HCC patients based on CT data regardless of tumor stage or applied therapy and compared both methods for superiority. Overall, deep-learning algorithms outperformed conventional radiomics features and could help to predict OS. Still, the clinical Cox-regression model showed the best performance in the presented setting.

To the best of our knowledge, our study is the first radiomics analysis of CT scans for OS of HCC patients across all tumor stages and common therapies. Previous studies have focused on specific therapies or tumor stages. In addition, analyses were often based on only one contrast phase and rarely used the combination of HCC and liver parenchyma.

To date, the predictive power of deep-learning algorithms on CT images for predicting OS of HCC patients has not been comprehensively evaluated. Wang et al. reported a C-index of 0.58 for patients undergoing stereotactic radiotherapy^27^. Better results were observed in patients who received a TACE alone (C-indices = 0.65 and 0.73) or a combination of TACE and sorafenib (C-index = 0.72)^28–30^. The C-indices of 0.63–0.71 obtained in the present study are in line with the listed values and thus show the potential for outcome prediction even in HCC patients receiving different therapeutic approaches, although no significant risk stratification was possible.

Conventional radiomics models for predicting OS of HCC patients have been evaluated more commonly so far. C-indices from literature range between 0.63–0.78 and 0.60–0.67 for HCC patients undergoing surgery or TACE, respectively^31–35^. However, validation on holdout or external datasets was not always performed and risk stratification was not always possible. Here, the majority of our conventional radiomics models showed a performance close to random prediction. With a C-index of 0.66, only the best model (*HCC_art*) showed a value comparable to the literature.

Each deep-learning image-based model outperformed its conventional radiomics counterpart with statistically significance for the venous liver model (*liver_ven*), leading us to the conclusion that deep-learning may offer an enhanced prognostic utility. The main reason for the limited performance of the conventional radiomics approach may be the heterogeneous study cohort. Previous studies reported lack of reproducibility of hand-crafted radiomics features between different CT scanners, acquisition and reconstruction parameters^8,36–39^. As we used CT data from 24 different scanners, acquisition parameters were heterogeneous, which may have negatively affected reproducibility of radiomics features. In contrast, features extracted from deep-learning may be more robust^40^. This observation aligns with findings from a comparative study on head and neck cancer OS prediction, which demonstrated that deep learning models exhibit superior generalizability across different institutions compared to conventional radiomics approaches^41^. Overall, the clinical model based on Cox-regression was superior to all imaging approaches with significantly different OS between the stratified risk groups suggesting a high importance of clinical factors for generalized prediction models. The parameters of the final signature, consisting of GGT, AFP and the HCC volume, have a known impact on the prognosis of affected patients. Elevated GGT levels may indicate liver damage, such as chronic hepatic parenchymal remodeling or HCC^42^, and are associated with OS in HCC^43,44^. AFP is the most common serum marker in HCC, with higher AFP levels associated with poorer OS^45,46^. HCC volume is associated with tumor malignancy and infiltrative behavior^47^. Wu et al. point out that tumor size at diagnosis is an independent prognostic factor for OS, irrespective of tumor grade, stage, or treatment selected^48^. Our results support these findings. However, other parameters, such as the MELD-Score were not included in the clinical model. Although this factor has been identified as predictor of HCC prognosis^49^, within the scope of our multi-step machine learning workflow and the heterogeneity of our patient cohort, the inclusion of additional clinical parameters did not give benefit to the predictive performance and its generalizability. As lack of clinical data continues to be a non-negligible problem in patient care in some cases, the development of image parameter-based deep-learning and conventional radiomics models is essential. Therefore, further studies in larger patient groups are essential to further explore the comparative potential between image-based algorithms and clinical models.

Interestingly, the clinical deep-learning model was outperformed by the clinical Cox-regression model, although the difference was not statistically significant. One potential explanation for this finding is that the clinical deep-learning model may not have been fully optimized. Specifically, overfitting on the development cohort was observed, which suggests that further refinement and optimization of the model hyperparameters may lead to a better performance on future datasets.

The complexity of deep-learning models raises questions about their value for OS prediction. To increase the value of deep-learning models for OS prediction, the models should be interpretable and easily to understand and rely on for physicians. To improve the interpretability of the deep-learning models, their output was correlated with the conventional radiomics features. For the HCC model, a high correlation with the HCC volume was observed (Spearman R = 0.94), indicating that an increase in HCC volume corresponds to lower OS. This finding is consistent with the results of the clinical Cox-regression model of this study. Similarly, predictions of the liver model were associated with liver volume (R = 0.86), suggesting that an increase in liver volume corresponds to lower OS. This finding contradicts the expectation that progressing cirrhosis is associated with a decreasing liver volume leading to lower OS^50^. Future research should aim to better understand the complex patterns that deep-learning algorithms can detect.

There are some limitations of this study. First, it was a retrospective study with a small sample size of 114 patients with limited follow-up duration. Especially for CNNs, the limited sample size increases the risk of overfitting and reduces result reliability. However, multiple strategies were employed to minimize the risk of overfitting despite the limited sample size: (i) early stopping of the training process using cross-validation; (ii) masking the CT image to the ROI to prevent overfitting on surrounding anatomical structures; (iii) architectural considerations like batch normalization, the use of dropout layers and pooling layers and data augmentation; (iv) ensemble prediction by averaging the output from 45 individually trained models for the final prediction. The small resulting differences of CNN performances between the development and validation cohort suggest that overfitting is unlikely, despite the limited sample size. Second, the study population has high heterogeneity in terms of various factors such as applied treatment, CT acquisition protocols, and HCC tumor stages. While this heterogeneous group reflects everyday clinical practice, it may also limit the generalizability of the findings, as the specific distribution of clinical characteristics and treatment approaches may differ significantly across clinics. In addition, 1/85 and 1/29 patients in the development and validation cohort, respectively, were expected to die from treatment-related causes. Due to this minority, the impact on the developed models can be considered negligible. Third, not all clinical parameters were available and only the largest lesion was segmented in multifocal HCC. Whole tumor burden analysis may improve the efficiency of OS prediction, although all HCC lesions were included as the entire liver parenchyma was additionally segmented. Fourth, in the deep-learning models, the analysis was conducted on the masked CT images, which may introduce bias towards the volume of the regarded ROI and could potentially exclude areas with prognostic value in the peritumoral region^51^. However, the utilization of masked images may also result in the reduction of background information. This compels the model to prioritize important areas within the image. As a result, this approach has the potential to enhance both the reliability and quality of the model’s outputs^52^. Moreover, this approach ensures that the conventional radiomics and deep-learning models are based on similar data, which enables a more equitable comparison. Fifth, all segmentations were derived manually by a single radiologist and subjective bias cannot be excluded. Therefore, further studies on a large sample size are needed to increase the reliability of the results.

In conclusion, deep-learning algorithms showed superiority over conventional radiomics for predicting OS in patients with HCC across a wide spectrum of therapies, tumor stages and CT acquisition protocols. In total, they showed comparable performance to previously presented models, which were, however, adjusted to therapy subgroups. The results advocate the development of deep-learning models in the clinical prognosis of HCC patient survival on a larger scale and may provide important information in the clinical setting, especially when clinical data is limited.

### Supplementary Information

Supplementary Information.

## Data Availability

The datasets used and analyzed during the current study are available from the corresponding author on reasonable request.
