# Supplementary material for: Comparative analysis of radiomics and deep-learning algorithms for survival prediction in hepatocellular carcinoma
Source: Sci Rep. 2024 Jan 5;14:590. doi: 10.1038/s41598-023-50451-3 (PMC10770355; doi:10.1038/s41598-023-50451-3)
Supplement: Supplementary file 1 — Supplementary Information. [file 41598_2023_50451_MOESM1_ESM.docx]

**Supplementary Information**

**Table S1**. CT acquisition and reconstruction settings for the development and validation cohort.

| **Acquisition parameter** | | **Development cohort  (n = 85)** | | **Validation cohort  (n = 29)** | |
| --- | --- | --- | --- | --- | --- |
|  |  | **art** | **ven** | **art** | **ven** |
| Manufacturer | GE MEDICAL SYSTEMS | 8 | 8 | 2 | 2 |
|  | Philips | 3 | 3 | 1 | 1 |
|  | SIEMENS | 70 | 70 | 26 | 26 |
|  | TOSHIBA | 4 | 4 | 0 | 0 |
| Scanner model | Aquilion | 2 | 2 | 0 | 0 |
|  | Aquilion PRIME | 2 | 2 | 0 | 0 |
|  | Biograph 16 | 1 | 2 | 0 | 0 |
|  | BrightSpeed | 4 | 3 | 0 | 0 |
|  | BrightSpeed S | 1 | 1 | 0 | 0 |
|  | Brilliance 16 | 1 | 1 | 0 | 0 |
|  | Brilliance 64 | 1 | 1 | 1 | 1 |
|  | Discovery CT750 HD | 0 | 0 | 1 | 1 |
|  | Emotion 16 | 1 | 1 | 0 | 0 |
|  | Emotion 16 (2007) | 3 | 3 | 2 | 2 |
|  | Emotion 6 (2007) | 1 | 1 | 1 | 1 |
|  | Emotion 6 (2010) | 1 | 1 | 0 | 0 |
|  | Optima CT520 Series | 2 | 2 | 1 | 1 |
|  | Optima CT660 | 1 | 1 | 0 | 0 |
|  | SOMATOM Definition AS | 5 | 5 | 1 | 1 |
|  | SOMATOM Definition AS+ | 30 | 30 | 13 | 13 |
|  | SOMATOM Definition Edge | 4 | 4 | 1 | 1 |
|  | SOMATOM Force | 12 | 12 | 1 | 1 |
|  | SOMATOM Scope | 0 | 0 | 1 | 1 |
|  | Sensation 16 | 5 | 5 | 2 | 2 |
|  | Sensation 40 | 1 | 1 | 0 | 0 |
|  | Sensation 64 | 5 | 5 | 4 | 4 |
|  | Spirit | 1 | 1 | 0 | 0 |
|  | iCT 256 | 1 | 1 | 0 | 0 |
| Tube voltage in kV | 70 | 1 | 0 | 0 | 0 |
|  | 80 | 4 | 1 | 1 | 0 |
|  | 90 | 4 | 5 | 0 | 0 |
|  | 100 | 26 | 28 | 9 | 10 |
|  | 110 | 3 | 2 | 3 | 2 |
|  | 120 | 41 | 42 | 14 | 14 |
|  | 130 | 5 | 5 | 1 | 2 |
|  | 140 | 1 | 2 | 1 | 1 |
| Pixel spacing x in mm | x < 0.6 | 1 | 0 | 0 | 0 |
|  | 0.6 <= x < 0.7 | 10 | 10 | 6 | 6 |
|  | 0.7 <= x < 0.8 | 37 | 39 | 10 | 9 |
|  | 0.8 <= x < 0.9 | 23 | 22 | 12 | 12 |
|  | 0.9 <= x < 1.0 | 14 | 14 | 1 | 2 |
| Slice thickness in mm | 1.25 | 2 | 2 | 0 | 0 |
|  | 2 | 2 | 1 | 1 | 0 |
|  | 2.5 | 3 | 1 | 0 | 0 |
|  | 3 | 60 | 58 | 19 | 19 |
|  | 3.75 | 2 | 2 | 2 | 1 |
|  | 5 | 16 | 21 | 7 | 9 |
| Reconstruction kernel | Missing information | 11 | 10 | 3 | 3 |
|  | B20f | 3 | 0 | 1 | 0 |
|  | B30f | 10 | 28 | 2 | 7 |
|  | B31f | 17 | 21 | 12 | 13 |
|  | B31s | 4 | 5 | 1 | 1 |
|  | B40f | 19 | 1 | 5 | 0 |
|  | B41f | 1 | 0 | 0 | 0 |
|  | B41s | 3 | 3 | 3 | 3 |
|  | Br36d | 3 | 5 | 0 | 0 |
|  | Br40d | 7 | 7 | 1 | 1 |
|  | Bv36d | 2 | 0 | 0 | 0 |
|  | FC08 | 2 | 2 | 0 | 0 |
|  | FC11 | 1 | 1 | 0 | 0 |
|  | FC17 | 1 | 1 | 0 | 0 |
|  | I30f | 0 | 1 | 1 | 1 |
|  | I31f | 1 | 0 | 0 | 0 |
| Contrast agent | Missing information | 60 | 60 | 23 | 23 |
|  | Accupaque | 1 | 1 | 1 | 1 |
|  | Solutrast | 2 | 2 | 0 | 0 |
|  | Imeron | 2 | 2 | 0 | 0 |
|  | Optiray | 1 | 1 | 0 | 0 |
|  | Ultravist | 19 | 19 | 5 | 5 |

**Abbreviations:** **art** - arterial phase; **CI** - confidence interval; **ven** - venous phase

**Table S2**. Settings used for image processing of the CT scans and feature computation.

| **Image interpolation** | Interpolation method | Cubic spline |
| --- | --- | --- |
|  | Voxel dimensions in mm³ | 1x1x1 |
|  | Anti-aliasing smoothing parameter | 0.98 |
| **ROI interpolation** | Interpolation method | Linear |
|  | Threshold | 0.5 |
| **Volume adaptation** | Cropping distance in mm | 150 |
| **ROI resegmentation** | Threshold in HU | Low: -200, high: 500 |
| **Feature extraction** | Discretization algorithm | Fixed_bin_number with 32 bins |
|  | Spatial calculation method | 3D |
|  | Merge method | Volume merge |
|  | Distance (in voxels) for GLCM and GLSZM | 1 |

**Abbreviations:** **GLCM** - gray level co-occurrence matrix; **GLSZM** - gray level size zone; **HU** - Hounsfield Units; **ROI** - region of interest

**Table S3**. Settings used for feature processing and model building.

| **Processing step** | **Method** | **Details** |
| --- | --- | --- |
| Missing value imputation | Median | Patient excluded if missing features > 30% |
| Feature transformation | Yeo-Johnson normalization, Z-standardization |  |
| Feature filtering | Univariate Regression | Features with p-values > 0.3 excluded |
| Feature clustering | Hierarchical Agglomerative Clustering (hclust) | Cluster linkage method: average  Cluster cut method: silhouette  Cluster similarity metric: mcfadden_r2  Threshold: 0.3  Cluster representation method: best_predictor |
| Feature importance | Minimum-Redundancy-Maximum-Relevance (MRMR) | Variable importance aggregation method: stability |
| Hyperparameter optimization | Sequential Model-Based Optimization (SMBO) | Model performance metric: concordance index  Optimization function: balanced  Hyperparameter learning: Gaussian_process  Maximum number of bootstraps for optimization: 20 |
| Feature Importance Evaluation | 15 x 3-fold Cross-Validation | top 5 occurrences in all 45 internal models |
| Model development | Cox proportional hazards model | Cross-validation signature size: 1-10  Radiomics model signature size: Median signature size of all 45 internal models |

**Table S4.** Hyperparameters for the deep-learning models.

| **Hyperparameters** | **Setting** |
| --- | --- |
| Optimization algorithm | Adam |
| Learning rate (image-based models) | 0.000001 |
| Learning rate (clinical model) | 0.0001 |
| Weight initialization | Default Pytorch Initialization |
| Data augmentation | Random translation of maximum 20 mm, Random rotation of maximum 3 degrees, both applied uniformly |
| Batch size | 16 |
| Activation function (hidden layers) | Leaky ReLU with 𝛼 = 0.1 |
| Activation function (output layer) | Sigmoid function |
| Padding (image-based models) | Same |
| 1st Convolutional Layer (image-based models) | Filter size: 5 × 5 × 5, Stride: 2 |
| 2nd Convolutional Layer (image-based models) | Filter size: 3 × 3 × 3, Stride: 2 |
| 3rd and 4th Convolutional Layers (image-based models) | Filter size: 3 × 3 × 3, Stride: 1 |
| Max Pooling Layers (image-based models) | Filter size: 2 × 2 × 2, Stride: 1 |
| Dropout probability (image-based models, Conv_Group,) | 0.4 |
| Dropout probability (image-based models, Dense_Group) | 0.25 |
| Dropout probability (clinical model) | 0.7 |

**Table S5**. Signatures of the supplementary conventional radiomics models and their respective multivariate Cox-regression parameters. The hazard ratio (HR) [95% CI] and the corresponding p-values of the regression are shown. * = statistically significant (p < 0.05)

| **Supplementary conventional models** | **Variables** | **Hazard Ratio [95% CI]** | **p-value** |
| --- | --- | --- | --- |
| *HCC_art* | szm_zs_entr_3d_fbn_n32_HCC_art | 1.37 [0.98-1.93] | 0.07 |
|  | cm_energy_d1_3d_v_mrg_fbn_n32_HCC_art | 0.88 [0.53-1.48] | 0.63 |
|  | rlm_lrhge_3d_v_mrg_fbn_n32_HCC_art | 1.64 [0.95-2.83] | 0.08 |
| *HCC_ven* | stat_skew_HCC_ven | 0.83 [0.62-1.11] | 0.21 |
|  | szm_zs_entr_3d_fbn_n32_HCC_ven | 1.20 [0.83-1.74] | 0.34 |
| *Liver_art* | szm_lzhge_3d_fbn_n32_liver_art | 1.52 [1.04-2.21] | 0.03* |
|  | morph_pca_least_axis_liver_art | 0.88 [0.41-1.83] | 0.73 |
|  | morph_area_mesh_liver_art | 1.31 [0.82-2.09] | 0.26 |
|  | dzm_zd_var_3d_fbn_n32_liver_art | 1.57 [0.80-3.10] | 0.19 |
|  | morph_vol_dens_conv_hull_liver_art | 0.75[0.53-1.07] | 0.11 |
| *Liver_ven* | ivh_v50_liver_ven | 0.73 [0.54-0.99] | 0.04* |
|  | morph_diam_liver_ven | 1.27 [0.90-1.90] | 0.18 |
|  | ih_min_grad_fbn_n32_liver_ven | 0.68 [0.48-0.96] | 0.03* |

**Abbreviations:** **art** - arterial phase; **CI** - confidence interval; **HCC** - hepatocellular carcinoma; **ven** - venous phase

**Table S6**. Model performances of the internal cross-validation for the conventional radiomics approach. C-indexes are indicated using the median [95% CI].

|  | | **Training fold C-index [95% CI]** | **Testing fold C-index [95% CI]** |
| --- | --- | --- | --- |
| **Primary conventional models** | Clinical model | 0.71 [0.61-0.78] | 0.59 [0.43-0.78] |
|  | Image-based model | 0.81 [0.70-0.87] | 0.52 [0.41-0.64] |
|  | Combined model | 0.83 [0.71-0.89] | 0.53 [0.34-0.71] |
| **Supplementary conventional models** | *HCC_art* | 0.65 [0.55-0.73] | 0.50 [0.39-0.62] |
|  | *HCC_ven* | 0.66 [0.54-0.75] | 0.46 [0.36-0.59] |
|  | *Liver_art* | 0.72 [0.58-0.81] | 0.53 [0.45-0.64] |
|  | *Liver_ven* | 0.73 [0.55-0.87] | 0.60 [0.50-0.86] |

**Abbreviations:** **art** - arterial phase; **CI** - confidence interval; **HCC** - hepatocellular carcinoma; **ven** - venous phase

**Table S7**. Model performances of the internal cross-validation for the deep-learning approach and the final number of training epochs. C-indexes are indicated using the median [95% CI].

|  | | **Training fold C-index [95% CI]** | **Testing fold C-index [95% CI]** | **Final number of epochs** |
| --- | --- | --- | --- | --- |
| **Primary Deep-Learning models** | Clinical model | 0.76 [0.70-0.82] | 0.54 [0.43-0.65] | 430 |
|  | Image-based model HCC (*HCC_art + HCC_ven*) | 0.59 [0.50-0.67] | 0.56 [0.44-0.68] | 39 |
|  | Image-based model Liver (*Liver_art + Liver_ven*) | 0.69 [0.62-0.77] | 0.55 [0.39-0.61] | 14 |
|  | Combined model | 0.69 [0.60-0.80] | 0.53 [0.32-0.75] | 383 |
| **Supplementary Deep-Learning models** | *HCC_art* | 0.53 [0.46-0.68] | 0.52 [0.36-0.67] | 136 |
|  | *HCC_ven* | 0.62 [0.50-0.68] | 0.56 [0.38-0.75] | 224 |
|  | *Liver_art* | 0.70 [0.61-0.78] | 0.57 [0.40-0.74] | 28 |
|  | *Liver_ven* | 0.64 [0.56-0.73] | 0.54 [0.32-0.69] | 19 |

**Abbreviations:** **art** - arterial phase; **CI** - confidence interval; **HCC** - hepatocellular carcinoma; **ven** - venous phase

**
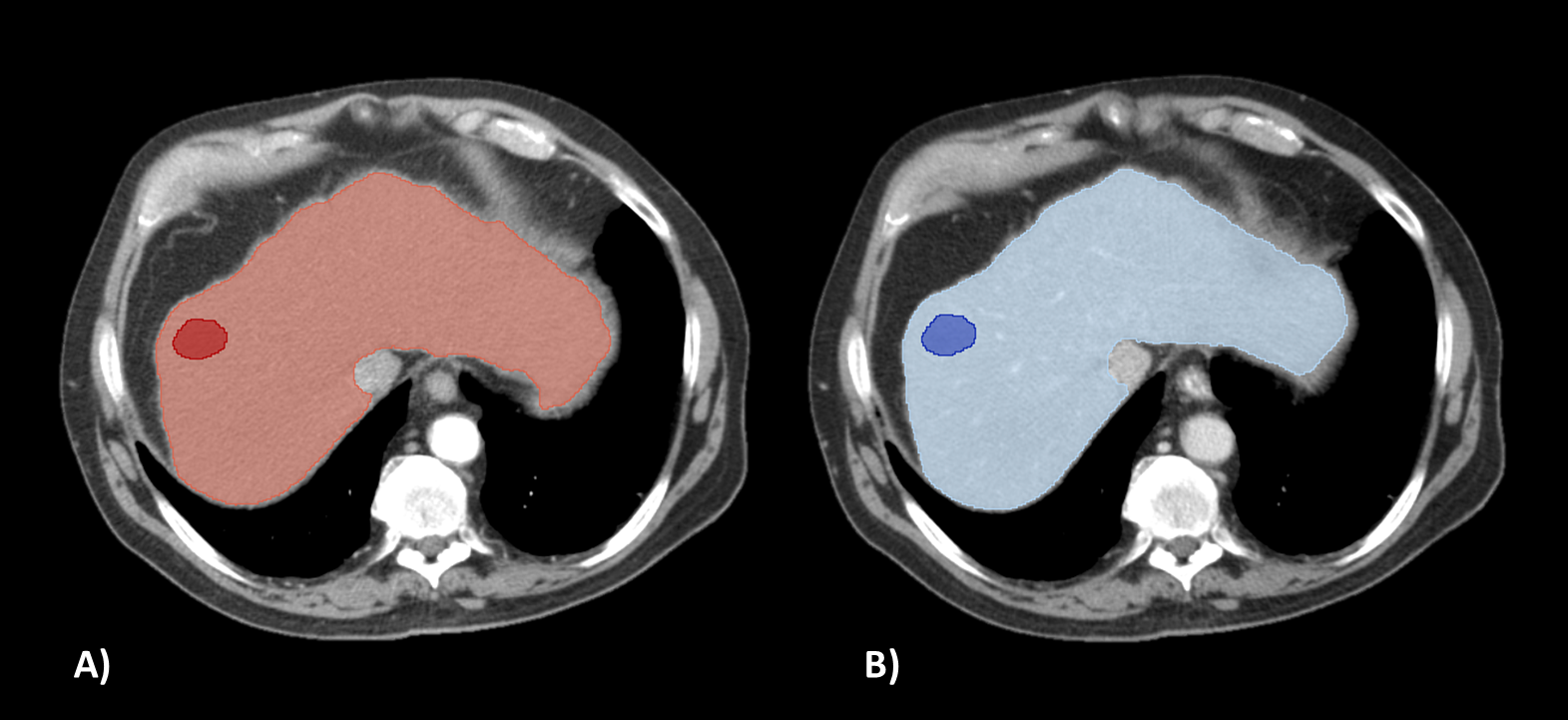
**

**Figure S1.** Exemplary segmentation of the liver parenchyma (light red and light blue) and the HCC (red and blue) in the arterial (A) and venous (B) contrast phase.


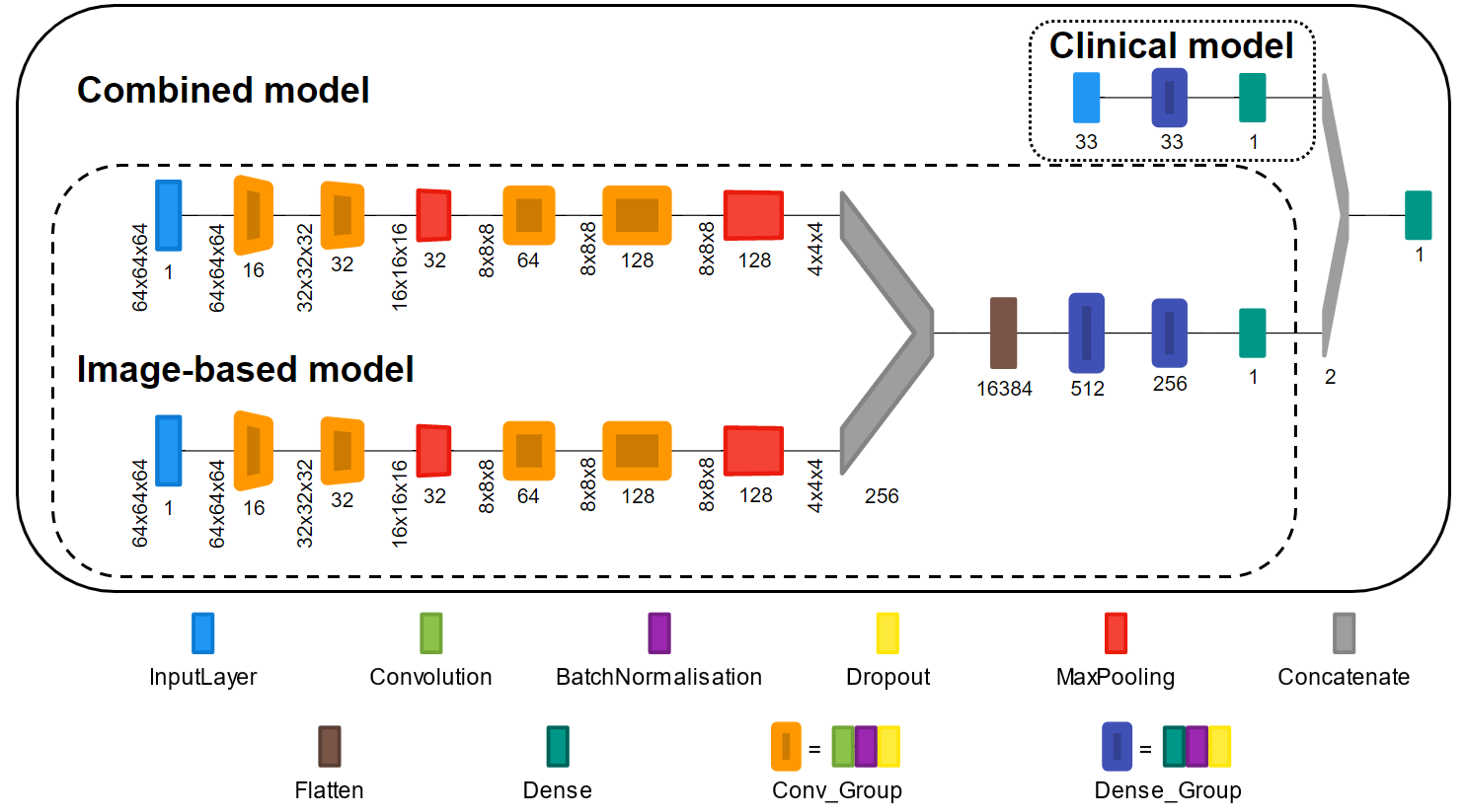


**Figure S2.** Illustration of primary deep-learning architectures. The combined model (solid line) consists of the concatenation of outputs from the image-based (dashed line) and clinical model (dotted line). The output layer is represented by the final dense layer in each network. The legend is presented at the bottom of the image. The dimension of the feature maps is shown vertically on the layers. The number of different feature maps (or features for dense layers) is provided below the layers. The model for the analysis of HCC is used for the image-based model.


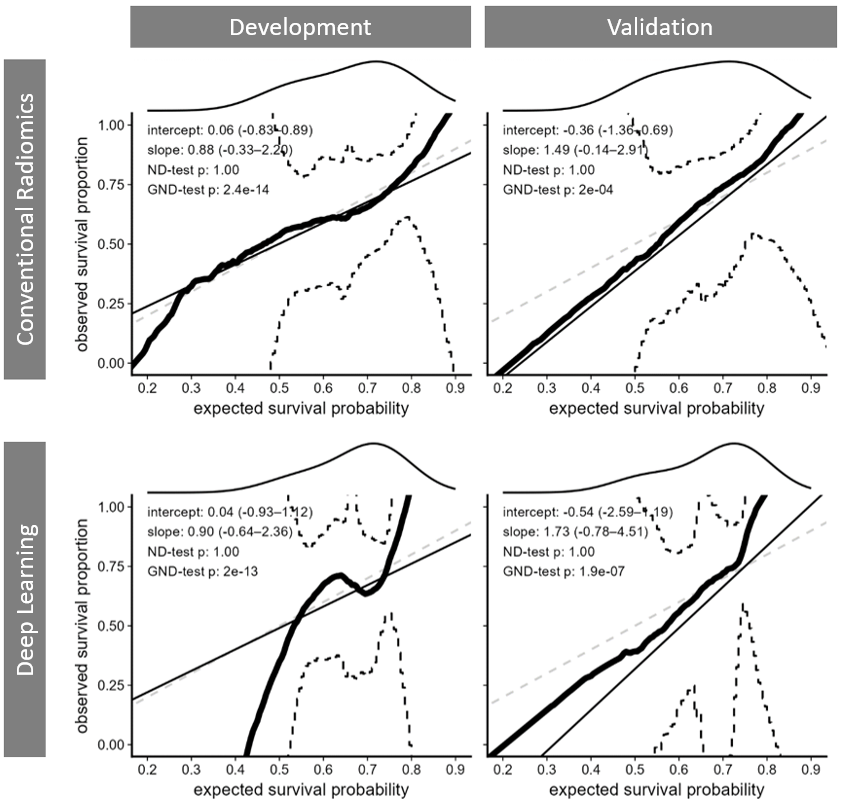

**Figure S3.** Calibration plots comparing conventional radiomics (upper panel) and deep learning (lower panel) models for HCC_art. Plots are divided into development (left) and validation (right) cohorts. The solid lines represent the calibration curves, indicating the relationship between expected and observed survival probabilities, while the dotted lines show the 95% confidence intervals. Each plot includes values for the intercept and slope, as well as p-values from the Nam-D'Agostino (ND) and Grønnesby and Borgan goodness-of-fit (GND) tests. The timepoint for the calibration plots was assessed at the median time-to-death of the development cohort (1.65 years).
